# Editorial to Special Issue Molecular Biology of Selenium in Health and Disease

**DOI:** 10.3390/ijms23020808

**Published:** 2022-01-12

**Authors:** Petra A. Tsuji, Dolph L. Hatfield

**Affiliations:** 1 Department of Biological Sciences, Towson University, 8000 York Road, Towson, MD 21252, USA; 2 Scientist Emeritus, Mouse Cancer Genetics Program, Center for Cancer Research, National Cancer Institute, National Institutes of Health, Bethesda, MD 20892, USA; hatfielddolph@gmail.com

The selenium field expanded at a rapid rate for about 45 years, from the mid-1970’s until about 2015 (see [1] for a summary of these 45 years). Then, the pace of major discoveries began to decline. However, we were fortunate enough to obtain many of the major players in the selenium field to write novel, up-to-date research articles and/or reviews on various topics of this fascinating element. Selenium is still regarded as one of the most interesting and health-beneficial elements. It will be fascinating to bear witness to additional discoveries in the field of selenium and selenoproteins in health and disease in the coming years.

There are 21 published manuscripts, including 13 research articles and 8 reviews, with over 120 different contributors, in this Special Issue, entitled “Molecular Biology of Selenium in Health and Disease”. Many of the most important subjects in the selenium field are covered. These include a historical perspective of the roles of selenium and selenoproteins in health and development, including its beneficial and detrimental aspects [2], the role of selenium in redox signaling in macrophages [3], as well as in bacterial and human selenoprotein biosynthesis [4], and its proposed cellular transport via a metal cation symporter [5]. Furthermore, selenium deficiency is discussed in the setting of cardiovascular function [6] and as a trigger for autoimmune diseases [7]. This Special Issue also provides insights into selenoprotein mechanisms, including SELENOF in prostate [8] and colon cancers [9], SELENOI in immune response [10], SELENOW’s interaction with thioredoxins [11], and SELENOP-mediated selenium transport in Hashimoto`s thyroiditis [12]. Furthermore, an overview of the pathogenic variants in selenoproteins genes from a population genomics perspective [13], a review on the human genetic disorders resulting in systemic selenoprotein deficiency [14], and a new strategy to assess the selenoproteome by non-radioactive isotopic labeling are presented [15]. This Special Issue also addresses the mechanistic aspects of selenoprotein synthesis, including the role of selenophosphate synthetase in endothelial cells [16] and mice [17], what is known about the mechanisms of UGA recoding and the fate of ribosomes that fail to incorporate selenocysteine [18], tRNA^[Ser]Sec^ isopentylation and its role in hypothalamic neurons [19], as well as the impact of a conditional tRNA^[Ser]Sec^ knockout on leptin sensitivity and weight gain in agouti-related, peptide-positive hypothalamic neurons [20]. Lastly, a review of selenium and viral infections, including HIV and also the ongoing COVID-19 pandemic [21], and a research article on the interactions between selenium, selenoproteins and HIV-1 replication in human CD4 T-lymphocytes [22], provide important considerations regarding this trace element’s impact on viral infections threatening human health.

When we were in the early stages of organizing this Special Issue on Molecular Biology of Selenium in Health and Disease, with the assistance of our internal editor at the *International Journal of Molecular Sciences*, Ms. Lachelle Fang, we asked her if we could dedicate this Special Issue to Professor (Prof.) Dr. Leopold Flohé, M.D., who made many important discoveries to the growth and expansion in the field of this unique element. We were delighted that Editor Fang informed us that we could. In addition, Ms. Fang was most helpful throughout the organization of our Special Issue by handling so much of the correspondence with authors, and by obtaining reviewers for each of the papers. We are certainly indebted to her.

This Special Issue on ‘Molecular Biology of Selenium in Health and Disease’ within the various *International Journal of Molecular Sciences* Special Issues, is dedicated to Prof. Dr. Leopold Flohé, M.D. (Figure 1) for his many contributions to the selenium field. His seminal discoveries have played important roles in developing the molecular biological aspects of selenium, particularly regarding the essential roles of selenium in health and the development of humans and many other higher lifeforms. These fundamental discoveries included the identity of the first selenium-containing protein, glutathione peroxidase in 1973 [23], a tetrameric selenoenzyme, designated glutathione peroxidase 1 (GPx1). The significance of this finding is regarded as providing the foundation of the selenium field [24], and it linked selenium to the underlying metabolism in humans and other mammals. Prof. Dr. Leopold Flohé’s work was largely responsible for the Food and Drug Administration recognizing selenium as a daily supplement for domestic animals in 1979 and for humans in 1981 [25]. Interestingly, almost 10 years passed before another selenoprotein was found in animals. Prof. Dr. Flohé, M.D. and his group had a major hand in identifying the second selenoenzyme in mammals, a monomeric selenoenzme, gluthathione peroxidase 4 (GPx4) [26,27]. Subsequently, GPx4 was found to be an essential selenoprotein in mammalian cell development, while GPx1 was found to be a stress-related or non-essential selenoprotein in mammalian cell development [28].

Prof. Dr. Flohé’s efforts were not limited to glutathione peroxidases. In the late 1990’s, he and his research team identified additional novel enzymes, tryparedoxin and tryparedoxin peroxidase, which are part of the hydroperoxide metabolism in trypanosomatids [29]. Dr. Flohé’s PhD student E. Nogoceke was awarded the ‘Paper of the Year’ award in 1998 for this work, leading to the discovery of the peroxidase system in trypanosomatids. Interestingly, tryparedoxin peroxidase is not only a homolog of the yeast thiol-dependent antioxidant protein discovered by Earl Stadtman in 1988, and was later found to be trypanosomatid’s thioredoxin peroxidase [30]. Dr. Flohé continued his research efforts in the field of redoxbiology, focusing on mitochondria as an important source of superoxides [31,32], and hydroperoxide metabolism in mycobacteria [24,33].

Prof. Dr. Flohé, M.D. has also won numerous prestigious awards and been presented with numerous distinguished honors. He was awarded the Fellowship of the Studienstiftung des Deutschen Volkes (German Academic Scholarship Foundation funded by the Federal Ministry of Education and Research) to support his education from 1962 to 1968, which is Germany’s oldest, largest, and most prestigious scholarship foundation. In 1973, he won the Award of the Anna-Monika-Foundation for the basic research he carried out on endogenous depression, and in 1985, he was presented the Claudius-Galenus Award for the production of urokinase by recombinant technology. Prof. Dr. Flohé, M.D. was presented with two superb awards in 1997, an Honorary Degree from the University of Buenos Aires, Argentina for his major achievements in parasitology, and the Klaus Schwarz Commemorative Medal for his pioneering work in research on the trace element selenium.

In 1998, Dr. Flohé was awarded the ‘Science and Humanity Prize’ for lifetime achievements by the Oxygen Club of California. A few years later, in 2006, he was awarded the Trevor Frank Slater Award and Gold Medal, which is the highest academic and research prize in the redox biology and medicine/free radical field conferred by the Society for Free Radical Research International, for lifetime achievements in science. 

Furthermore, in 2010, he was recognized as a Redox Pioneer, an award given to authors whose publications on redox biology have been cited more than 1,000 times, and who have over 20 articles that have been cited more than 100 times [24]. A few years ago, in his honor, the Leopold Flohé Redox Pioneer Young Investigator Award was created by the Society for Free Radical Research International, which selects a scientist below the age of 45 years with “outstanding novel findings in the field of biological redox processes, working already independently, and leading an own group of young scientists having published high quality papers” (https://www.sfrr-europe.org/index.php/awards/leopold-flohe-award, accessed on 11 January 2022). Lastly, in 2019, he was nominated as an honorary member of the international Scientific Network “Selenium Sulfur Redox and Catalysis” (SeSRedCat). It is indeed a pleasure and an honor to dedicate this Special Issue of the *IJMS* to Prof. Dr. Leopold Flohé, M.D.

## Figures and Tables

**Figure 1:**
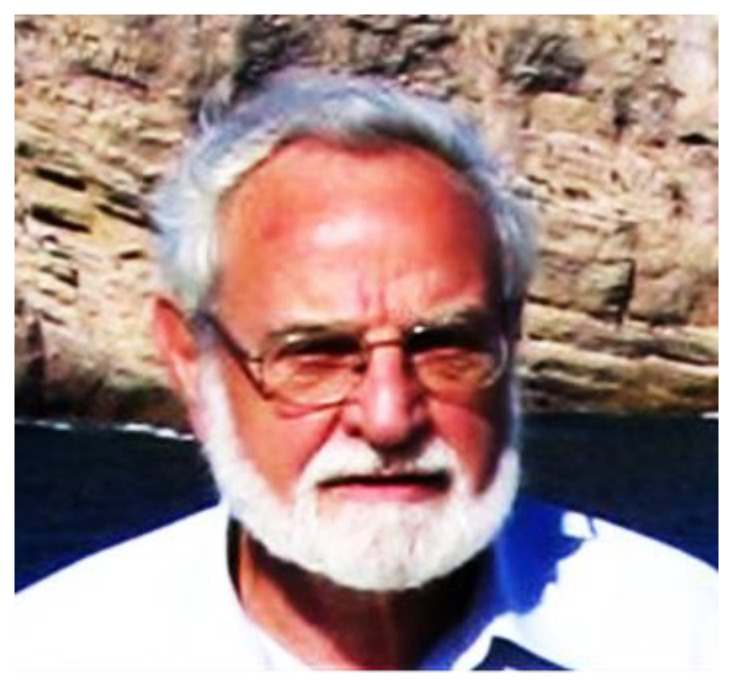
Photograph of Prof. Dr. Leopold Flohé, M.D (taken from his LinkedIn profile at https://www.linkedin.com/in/leopold-flohé-66b6239b, accessed on 22 December 2021).
